# Cadherin CsCad plays differential functional roles in Cry1Ab and Cry1C intoxication in *Chilo suppressalis*

**DOI:** 10.1038/s41598-019-44451-5

**Published:** 2019-06-11

**Authors:** Lixiao Du, Geng Chen, Lanzhi Han, Yufa Peng

**Affiliations:** State Key Laboratory for Biology of Plant Diseases and Insect Pests, Institute of Plant Protection, Chinese Academy of Agricultural Sciences, 100193 Beijing, China

**Keywords:** RNAi, Entomology, RNAi, Entomology

## Abstract

Transgenic rice lines expressing *Bacillus thuringiensis* (Bt) toxins have been successfully developed for the control of *Chilo suppressalis*. However, the evolution of insect resistance is a major threat to Bt rice durability. Bt toxins function by binding specific receptors in the midgut of target insects; specifically, cadherin proteins have been identified as Cry toxin receptors in diverse lepidopteran species. Here, we report the functional roles of cadherin CsCad in the midgut of *C*. *suppressalis* in Cry1Ab and Cry1C toxicity. We expressed a recombinant truncated CsCad peptide (CsCad-CR11-MPED) in *Escherichia coli* that included the eleventh cadherin repeat and MPED region. Based on ligand blotting and ELISA binding assays, the CsCad-CR11-MPED peptide specifically bound Cry1Ab with high affinity but weakly bound Cry1C. The CsCad-CR11-MPED peptide significantly enhanced the susceptibility of *C*. *suppressalis* larvae to Cry1Ab but not Cry1C. Furthermore, the knockdown of endogenous CsCad with Stealth siRNA reduced *C*. *suppressalis* larval susceptibility to Cry1Ab but not Cry1C, suggesting that CsCad plays differential functional roles in Cry1Ab and Cry1C intoxication in *C*. *suppressalis*. This information directly enhances our understanding of the potential resistance mechanisms of *C*. *suppressalis* against Bt toxins and may assist in the development of effective strategies for delaying insect resistance.

## Introduction

The bacterium *Bacillus thuringiensis* (*Bt*) produces crystalline (Cry) proteins that exhibit specific insecticidal activity; thus, these proteins have been used worldwide in insecticidal spray formulations or produced in transgenic *Bt* crops^1^. These Cry toxins target midgut epithelial cells in susceptible insects to disrupt the epithelial barrier and allow the onset of septicaemia, which ultimately kills the insect^2^. Although the mechanism by which Cry toxins induce enterocyte death remains controversial^3^, the interaction between Cry toxins and insect midgut epithelial receptors is considered a critical determinant of toxin specificity and insect resistance^4^. All proposed models of Cry intoxication posit that cadherin plays a central role as Cry toxin receptor. In the most accepted model, the binding to cadherin leads to further proteolytic cleavage of the toxin and the formation of oligomers that then bind proteins tethered to the cell membrane via glycosylphosphatidylinositol anchors, resulting in oligomer insertion into the membrane to form pores^5^. An alternative model in which binding to cadherin triggers an intracellular cell death pathway has been proposed^6^.

Regardless of the model, different lines of evidence support that insect cadherin plays a critical role as a receptor in Bt intoxication in at least eight lepidopteran species^1^. The predicted structure of Cry-binding lepidopteran cadherins includes an amino-terminal signal peptide, 8–12 cadherin repeats (CRs), a membrane-proximal extracellular domain (MPED), a transmembrane domain, and a cytoplasmic domain. In lepidopterans, most Cry toxin-binding sites are located at or near the membrane-proximal cadherin repeats^5,7^. Cadherin fragments containing the critical toxin-binding region enhance the activities of Cry toxins in some lepidopterans^8–13^. However, cadherin fragments have also been reported to reduce Cry1A toxicity to some lepidopterans^14–17^. Moreover, mutations in the lepidopteran cadherin genes are genetically linked to resistance to Cry toxins in *Heliothis virescens*^18^, *Helicoverpa armigera*^19^, and *Pectinophora gossypiella*^10^. Furthermore, the downregulation of a cadherin gene is associated with resistance to Cry1Ab in *Diatraea saccharalis*^20^.

In contrast to the Cry1A model, the Cry1C mode of action has not been described in detail. Aminopeptidase N has been identified as an important receptor of Cry1C in *Spodoptera littoralis* by RNA interference *in vivo*^21^ and *in vitro*^22,23^. Moreover, a *S*. *exigua* Cry1C-resistant strain lacks SeAPN1 expression^24^. However, SeCad1b cadherin from *S*. *exigua* has also been identified as a receptor for Cry1C based on the enhanced Cry1C toxicity by a truncated SeCad1b peptide and reduced susceptibility to the toxin following the silencing of SeCad1 expression^25^.

The striped stem borer, *Chilo suppressalis*, is a major lepidopteran rice pest that is widely distributed in all rice-growing areas in China. While chemical control has been the most common method used to control this pest, the efficacy of pesticides has rapidly declined due to the evolution of resistance^26^. Transgenic rice expressing the Cry1A or Cry1C insecticidal proteins have been introduced as an environmentally sound alternative for the control of *C*. *suppressalis* in China^27^; however, the development of resistance to Bt rice in *C*. *suppressalis* is considered the most important threat to the future of this technology^28^. Due to the crucial role played by toxin receptors in susceptibility and resistance^29^, the identification of relevant Cry1A or Cry1C receptors is needed for the development of effective strategies delaying *C*. *suppressalis* resistance and designing of alternative Bt products targeting this pest. The binding of the Cry1A toxin to *C*. *suppressalis* aminopeptidase (CsAPN) and cadherin (CsCad) expressed in insect cell cultures suggests that these proteins play a role in Cry intoxication^30,31^. The knockdown of two cadherin genes (CsCad1 and CsCad2) reduced the susceptibility of *C*. *suppressalis* to Cry1C, suggesting that potential interactions exist between CsCad1 or CsCad2 and Bt toxin^32^. Despite the available evidence supporting the toxin interaction, the functional Cry1A or Cry1C receptor role of cadherin in *C*. *suppressalis* has not been demonstrated. To address this knowledge gap, in this study, we conducted a functional analysis using a truncated CsCad peptide expressed in bacteria and silencing the expression of endogenous CsCad by Stealth siRNA. The exposure to the heterologous CsCad-CR11-MPED peptide significantly enhanced the susceptibility of *C*. *suppressalis* larvae to Cry1Ab but not to Cry1C. The knockdown of CsCad enhanced the tolerance of *C*. *suppressalis* larvae to Cry1Ab but did not change their susceptibility to Cry1C. The result demonstrate that CsCad plays differential functional roles in Cry1Ab and Cry1C intoxication in *C*. *suppressalis*. This information is important to understand the potential resistance mechanisms of *C*. *suppressalis* to Bt toxins and will help to develop effective strategies for delaying insect resistance to Bt rice.

## Results

### Stage- and tissue-specific expression profiles of *CsCad*

**Figure 1:**
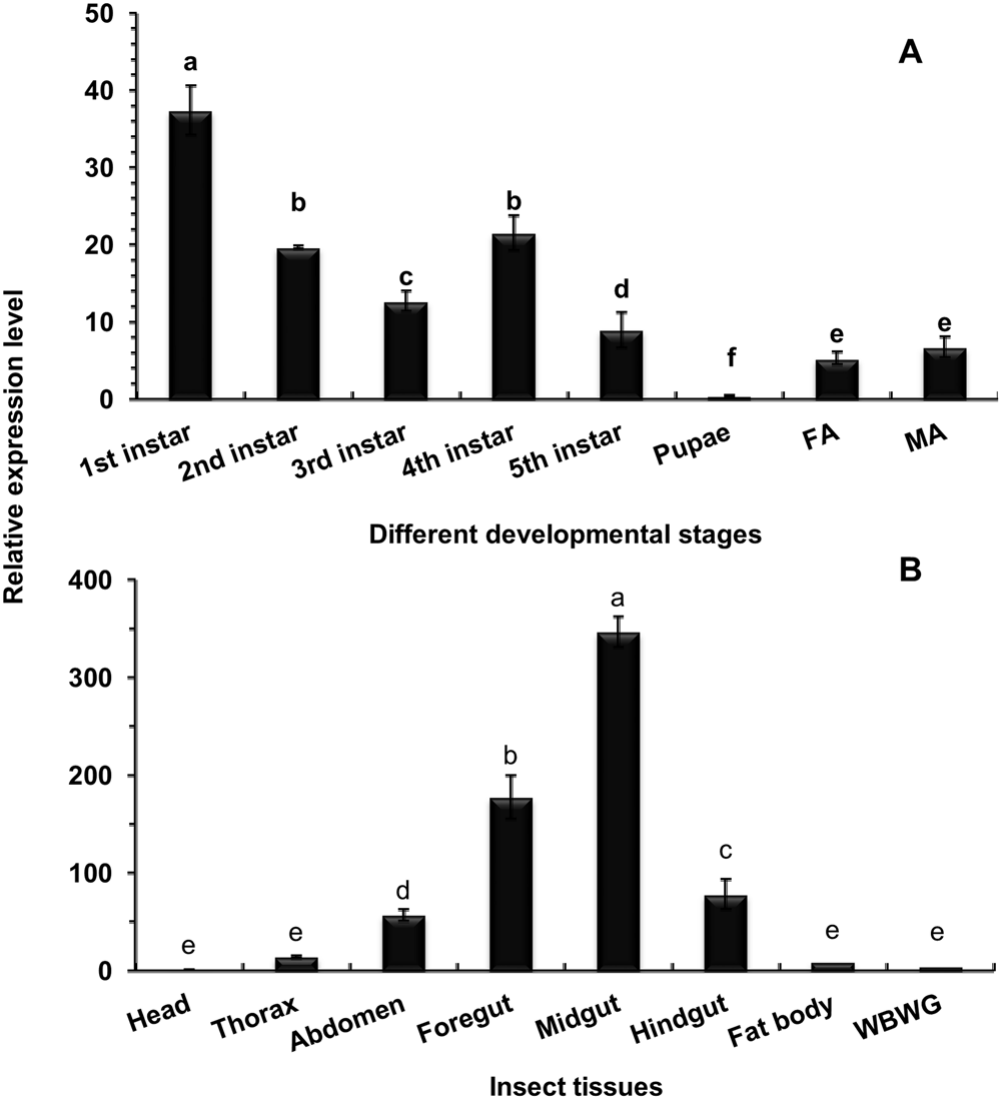
Expression profiles of *CsCad* genes in different larval tissues during different developmental stages of *C*. *suppressalis*. (**A**) Developmental stages included 1^st^, 2^nd^, 3^rd^, 4^th^, and 5^th^ instar larvae, pupae, and female (FA) and male (MA) adults. (**B**) Larval tissues included the head, thorax, abdomen, foregut (FG), midgut (MG), hindgut (HG), fat body, and whole body without gut tissue (WBWG). The mean ± SE was calculated to measure the relative transcript levels by the 2^−ΔΔCT^ method using the geometric mean of the β-Actin and *EF* genes for normalization. Standard errors of the mean were determined from three biological replicates, and each replicate included three technical replicates. Different letters on the top of the bars represent significant differences (*P* ≤ 0.05, Holm-Sidak test).

The expression of the *CsCad* gene was significantly higher in the first-instar larvae, followed by the 2nd- and 4th-instar larvae (Holm-Sidak test, *P* < 0.05). The lowest transcript levels of *CsCad* were detected in the pupal adults. *CsCad* was scarcely expressed during the adult stage (Holm-Sidak test, *P* < 0.05) (Fig. 1A).

The tissue-specific expression of *CsCad* was also tested. *CsCad* was more highly expressed in the MG than in the HG and FG (Holm-Sidak test, *P* < 0.05), and lower transcript levels were detected in the abdomen and thorax. The lowest *CsCad* expression levels were detected in the head, fat body and WBWG tissue (Holm-Sidak test, *P* < 0.05) (Fig. 1B).

### Ligand blotting of CsCad-CR11-MPED peptide with Cry1Ab or Cry1C

**Figure 2:**
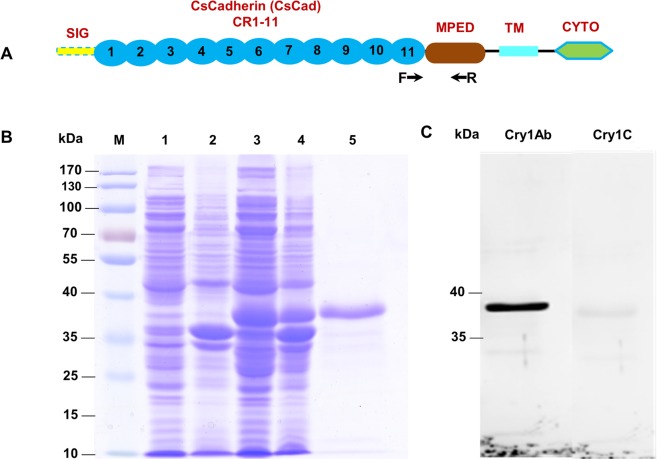
SDS-PAGE and ligand blot analysis of the expressed CsCad-CR11-MPED peptide fragment. (**A**) CsCad conserved domain structure including signal peptide (SIG), 11 cadherin repeats (CR11), membrane-proximal extracellular domain (MPED), transmembrane domain (TM) and cytoplasmic domain (CYTO). The arrows indicate the locations of the primers used to obtain the cadherin peptide fragment in the cadherin sequences. (**B**) Expressed and purified proteins were Coomassie-stained following 10% SDS-PAGE. Lane M: protein marker; Lane 1: 0 h IPTG-induced supernatant of Rosetta cells; Lane 2: 0 h IPTG-induced inclusion body; Lane 3: 6 h IPTG-induced soluble protein; Lane 4: 6 h IPTG-induced inclusion body; Lane 5: purified CsCad-CR11-MPED peptide from IPTG-induced inclusion body. (**C**) Purified CsCad-CR11-MPED peptide (2.0 µg) transferred to a PVDF membrane was probed with activated Cry1Ab or Cry1C toxin and detected using a polyclonal Cry1Ab or Cry1C antibody.

The purity of the recombinant CsCad-CR11-MPED peptide (Fig. 2A) was evaluated by 10% SDS-PAGE. A peptide of the expected size (~38-kDa) containing a His-tag (Fig. 2B) was confirmed. The ligand blot analysis indicated that the CsCad-CR11-MPED peptide bound the Cry1Ab toxin, while weaker binding to the Cry1C toxin was detected (Fig. 2C).

### Specific binding of the CsCad-CR11-MPED peptide to Cry1Ab or Cry1C

**Figure 3:**
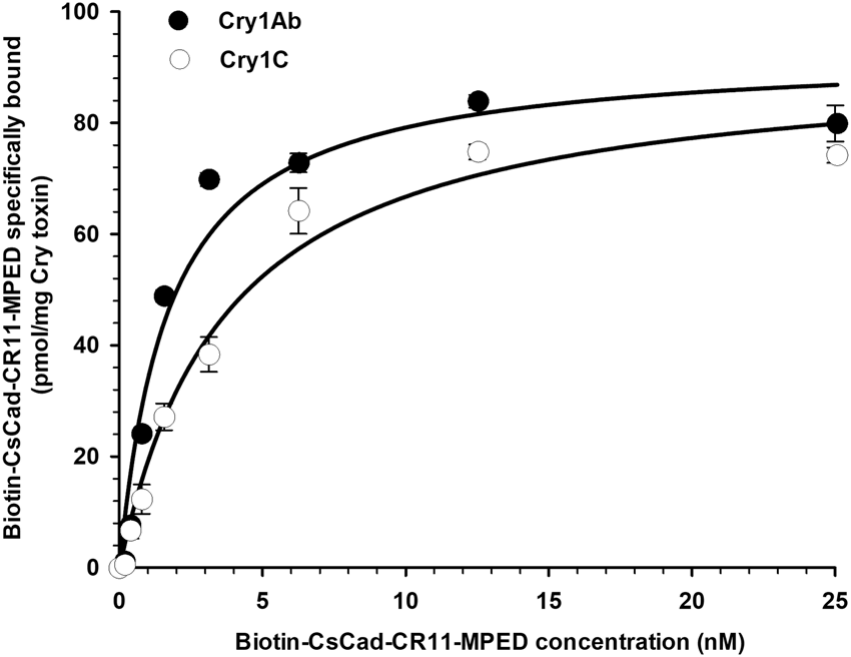
Binding saturation of CsCad-CR11-MPED peptide to Cry1Ab or Cry1C in ELISA binding assays. Microtitre plates coated with 1 µg of trypsinized Cry1Ab or Cry1C per well were incubated with increasing concentrations of biotinylated CsCad-CR11-MPED peptide alone or with a 1000-fold molar excess of unlabelled homologous peptide to determine the specific binding. Specific binding of the biotinylated CsCad-CR11-MPED peptide to Cry1Ab or Cry1C was calculated by subtracting the non-specific binding from the total binding in the absence of an unlabelled competitor. Each data point represents the mean of the results of two independent experiments performed in triplicate. Error bars show the standard deviations. Binding affinities (*K*_*d*_) were calculated based on specifically bound biotinylated peptides using a one-site saturation binding equation.

The biotin-labelled CsCad-CR11-MPED peptide specifically and saturably bound Cry1Ab or Cry1C in the ELISA assays as shown in Table 1 and Fig. 3. Using a one-site saturation binding model, an apparent dissociation constant (*K*_*d*_) of 1.72 ± 0.43 nM was calculated for CsCad-CR11-MPED peptide binding Cry1Ab (Table 1, Fig. 3). In contrast, a significantly lower binding affinity (t-test, *P* < 0.05) was observed for biotin-labelled CsCad-CR11-MPED binding Cry1C (*K*_*d*_ = 3.75 ± 0.71) (Table 1, Fig. 3). No significant differences (t-test, *P* > 0.05) were observed in the estimates of the binding site concentration (*B*_*max*_) of the Cry1Ab and Cry1C toxins and the CsCad1-CR11-MPED peptide (Table 1).

**Table 1:** Apparent dissociation constant (*K*_*d*_) and concentration of binding sites (*B*_*max*_) calculated from Cry1Ab or Cry1C toxin saturation binding assays using expressed and purified CsCad-CR11-MPED peptide.

Bt species	Binding constant (mean ± SE)	
	*K*_*d*_ ± SE^a^ (nM)	*B*_*max*_ ± SE (pmol/mg Cry toxin)
Cry1Ab	1.72 ± 0.43	92.80 ± 6.42
Cry1C	3.75 ± 0.71	91.81 ± 5.83

^a^SE = standard error of the mean.

### Effects of the CsCad-CR11-MPED peptide on the toxicity of Cry1Ab or Cry1C to *C*. *suppressalis* larvae

**Figure 4:**
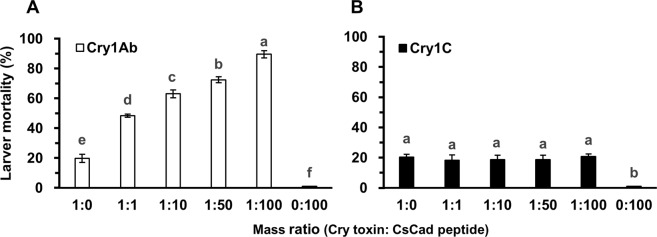
Synergistic effects of the CsCad-CR11-MPED peptide on Bt Cry1Ab (**A**) or Cry1C (**B**) toxicity. LC_20_ doses of Cry1Ab (0.25 µg/ml) or Cry1C (0.35 µg/ml) were used alone or with the expressed CsCad-CR11-MPED peptide in various toxin/peptide mass ratios. Each bioassay was conducted with 48 first-instar larvae per replicate and three replicates per concentration. Larval mortalities were recorded after 6 days of testing. Each data point represents the mean ± SE. Different lowercase letters above the bars show significant differences in larval mortalities among the various mass ratios of Cry1Ab or Cry1C and the CsCad-CR11-MPED peptide (Holm-Sidak test, *P* < 0.05).

We performed a bioassay to determine whether the CsCad-CR11-MPED peptide could enhance Cry1Ab or Cry1C toxicity to *C*. *suppressalis* larvae. The peptide significantly increased the mortality (Holm-Sidak test, *P* < 0.05) of Cry1Ab from 19.8% (toxin alone, LC_20_ dose) to 48.4% (1:1 mass ratio), 63.0% (1:10 mass ratio), 72.4% (1:50 mass ratio) and 89.6% (1:100 mass ratio), while no toxicity was detected in the bioassays with the peptide alone (Fig. 4A), suggesting that the peptide dose-dependently enhanced the toxicity of Cry1Ab to the *C*. *suppressalis* larvae. In contrast, the CsCad-CR11-MPED peptide did not significantly affect the Cry1C toxicity in the *C*. *suppressalis* larvae at any mass ratio (Holm-Sidak test, *P* > 0.05) (Fig. 4B).

### RNA interference (RNAi) silencing the expression of *CsCad*

**Figure 5:**
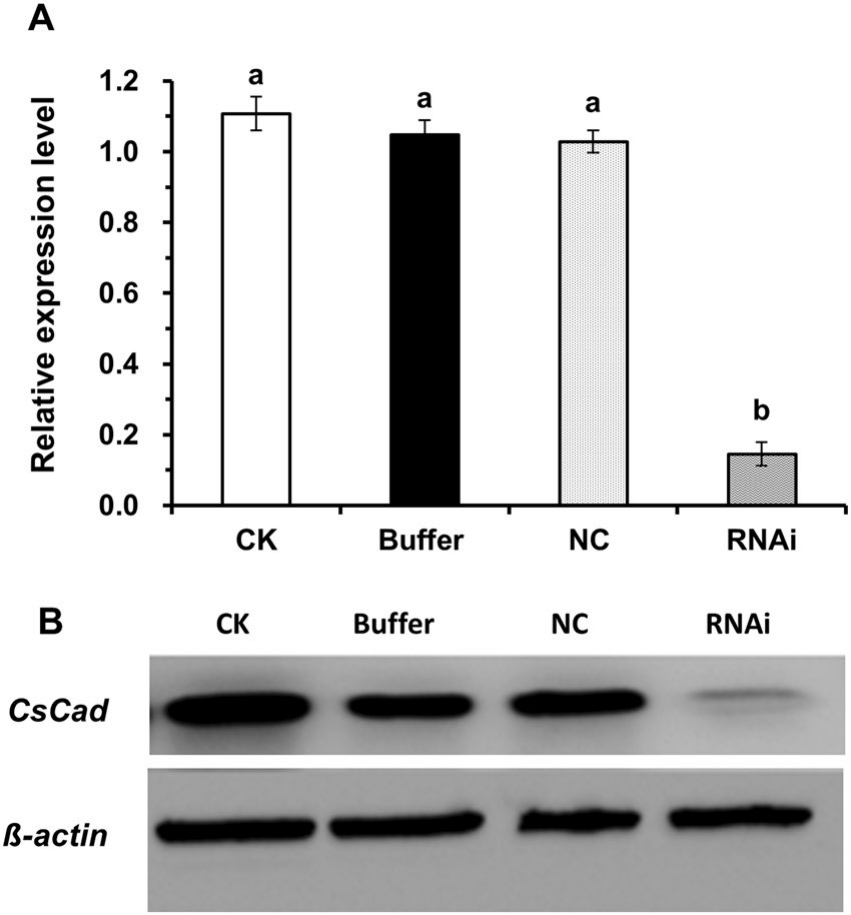
Knockdown of the *CsCad* gene in *C*. *suppressalis* larvae by oral RNAi delivery. (**A**) Transcript levels of *CsCad* were detected by qRT-PCR in silenced larvae fed Stealth siRNA (RNAi) relative to control non-silenced larvae treated with RNA dilution buffer (Buffer) and larvae silenced with Stealth siRNA negative control duplexes (NC). Each data point represents the mean ± SE, and different lowercase letters above the bars show significant differences (Holm-Sidak test, *P* < 0.05) in the relative expression levels among the different treatments at the same testing time after Stealth siRNA feeding. (**B**) Immunodetection of CsCad using polyclonal anti-CsCad serum in Stealth siRNA-fed (RNAi) and control larvae (CK, Buffer and NC) 72 h post-feeding. Each lane was loaded with an equal amount (10 µg) of protein based on the Bradford Protein Assay.

The oral delivery of siRNAs targeting the *CsCad* gene resulted in significantly reduced transcript levels (46.9% reduction, Holm-Sidak test, *P* < 0.05) as early as 48 h post-feeding. However, the lowest transcript level of *CsCad* was detected 72 h post-feeding when the amount of *CsCad* transcript in the larvae was reduced by approximately 85% compared to that in the control larvae (Holm-Sidak test, *P* < 0.05) (Fig. 5A). No significant differences in the *CsCad* transcript levels were found among the three control treatments (Fig. 5A). Accordingly, a significant reduction in the CsCad protein levels was also observed by a Western blot analysis of silenced first-instar larvae following the oral delivery of Stealth siRNA (Fig. 5B).

**Figure 6:**
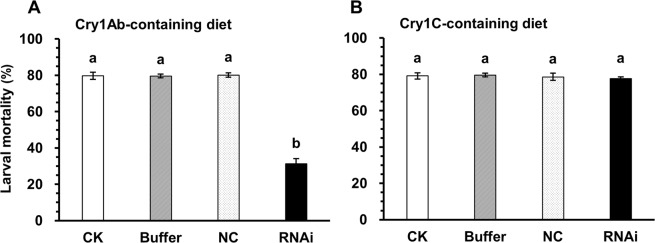
Toxicity of Cry1Ab (**A**) or Cry1C (**B**) to *C*. *suppressalis* larvae after Stealth siRNA feeding. Larval mortalities of silenced neonates (RNAi), non-silenced neonates (Buffer), and negative control neonates (NC) were determined after exposure to a diet containing activated Cry1Ab (1.25 µg/g in LC_80_ dose) or Cry1C (1.35 µg/g in LC_80_ dose) for 6 days. Each data point represents the mean ± SE. Different lowercase letters above the bars show significant differences (Holm-Sidak test, *P* < 0.05) in larval mortalities among the different treatments tested while fed a Cry1Ab- or Cry1C-containing diet.

In the bioassays, the Cry1Ab-induced mortality was significantly reduced in first-instar larvae exposed to Stealth siRNA against *CsCad* after 6 days of testing (Holm-Sidak test, *P* < 0.05) relative to the mortality observed in the controls. Larval mortality due to Cry1Ab decreased from 79.7% in the controls to 31.3% after 6 days in the larvae fed Stealth siRNA (Fig. 6A). However, we were unable to detect significant differences in the Cry1C-induced mortality between the control and Stealth siRNA-fed larvae (Fig. 6B).

## Discussion

Cadherins belong to a large and diverse family of glycosylated proteins that are usually anchored to the membrane by a transmembrane domain, and these proteins play critical roles in many cellular processes in vertebrates and invertebrates, including cell adhesion, migration, cytoskeletal organization and morphogenesis^7^. To date, cadherin has been studied in insects extensively as putative Bt toxin receptors. In lepidopterans, coleopterans and dipterans, many cadherins have been identified as receptors of Bt toxins, facilitating a post-binding specific proteolytic cleavage step that induces toxin oligomerization and pore formation and further mediates toxin susceptibility^5^.

In the current study, we identified CsCad cadherin in the MG of *C*. *suppressalis*, which showed the typical protein domains described in functional Cry toxin receptor cadherins^7^. Moreover, we observed that the transcription of CsCad was mainly localized to MG tissue and restricted to the larval developmental stages. The interactions between CsCad and Cry1Ab or Cry1C were detected using a CsCad-CR11-MPED peptide, which could enhance the toxicity of Cry1Ab but not Cry1C. In agreement with this observation, reducing the expression of CsCad by RNA interference led to a decrease in the susceptibility to Cry1Ab in *C*. *suppressalis*. However, the presence of CsCad did not enhance the Cry1C toxicity, and reducing the CsCad transcript levels did not cause obvious changes in the susceptibility to Cry1C in *C*. *suppressalis*. Therefore, these data support that CsCad plays differential functional role in the mode of action of Cry1Ab and Cry1C in *C*. *suppressalis*.

**Figure 7:**
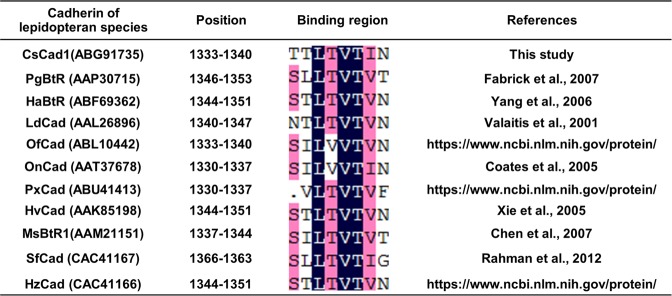
Alignment of Cry toxin-binding motifs in lepidopteran species cadherins.

Based on an analysis of previous references, insect cadherin proteins with a putative Cry receptor function have a high-affinity toxin-binding site in the CR region nearest to the plasma membrane^9,10,14,16,33,34^. In this study, we identified a putative Cry1A-binding region with an amino acid sequence similar to that identified in lepidopteran cadherin (Fig. 7). The CsCad-CR11-MPED of CsCad, which bound Cry1Ab with a high affinity and synergized Cry1Ab toxicity to *C*. *suppressalis* larvae, has an amino acid sequence that is similar to that of predicted Cry1A binding sites in cadherins from *S*. *frugiperda*^35^, *Manduca sexta*^9^, *H*. *virescens*^16^, *P*. *gossypiella*^10^ and other species^36–38^ (Fig. 7). Presumably, the amino acid sequences in Cry toxins bind cadherin via hydropathic complementarity^15^. The amino acid hydrophobicity pattern in the putative binding site of CsCad CR11-MPED is similar to that in known binding sites of cadherins in lepidopteran species, including SfCad, PgCad, HaCad, LdCad, PxCad, HvCad, MsCad and HzCad (Fig. 7).

Cadherin receptor fragments containing critical toxin-binding regions can enhance Cry toxin activity in lepidopterans^9,11,12,25,39^, coleopterans^33,40,41^ and dipterans^42,43^. Consistent with these results, we found that the Cry1Ab toxicity was significantly increased by the heterologous expression of CsCad CR11-MPED. However, the expression of soluble toxin-binding cadherin peptides has been reported to reduce Cry1A toxicity in *M*. *sexta*, *H*. *armigera* and *Bombyx mori*^11,14,16,17^. Presumably, the reduction in toxicity was due to the native conformation of the soluble toxin-binding cadherin fragment blocking the amino acid residues of the Cry1A toxin that bind receptors. In contrast, it is possible that an unfolded toxin-binding inclusion body cadherin with more exposed amino acid residues could modify its interactions with Cry1A toxins and other molecules on the insect MG epithelium and may be responsible for toxin synergism^11,17^. Further research has shown that the formation of a Cry1A toxin oligomer is partially responsible for the enhancement in Cry1A toxicity by cadherin fragments^11^. Another possibility is that different protein ratios of Cry1A toxins and cadherin competitors could have different effects, i.e., either inhibiting or enhancing toxicity^11^. In addition, the differences in the cadherin sequences used to design these fragments may account for the observed differences. However, the sequences of synergistic and competing cadherin fragments overlap, and the cause of the discrepancy in the activities of these peptides is unclear^17^.

Cadherins have been previously identified as putative Cry1A toxin-binding receptors on MG epithelial cells in many lepidopteran insects by RNA interference, although not in *C*. *suppressalis*. Previously, the binding of the Cry1Ab toxin to CsCad protein expressed in insect cell cultures preliminarily suggested that a functional interaction exists between Cry1Ab and CsCad in *C*. *suppressalis*^30^. In this study, we found a positive correlation between reduced CsCad transcript levels (an approximately 85% reduction) and decreased susceptibility (48.4% decrease in mortality) to Cry1Ab, providing strong evidence supporting the functional role of CsCad in Cry1Ab intoxication in *C*. *suppressalis*. Similarly, the specificity and functional role of Cry1Ac in conjunction with the *HaAPN1* gene have been illuminated by Sf21 cell-based assays and specific RNAi in both cell lines and whole insects^44^. Additionally, RNA interference knockdown of *DsCad1* led to a decrease in the susceptibility to Cry1Ab in *D*. *saccharalis*^20^. Moreover, Cry1Ab resistance in *D*. *saccharalis* is probably associated with the reduced expression of *DsCad*^20^. Therefore, the results obtained in this study strongly suggest that due to the specific binding of CsCad to the Cry toxin, CsCad represents a functional Cry1Ab receptor in *C*. *suppressalis*.

Our previous research showed that in the MG BBMV of *C*. *suppressalis*, Cry1A could bind proteins of approximately 15, 75, 115, and 200 kDa (cadherin) in size, while Cry1C recognized only four proteins less than 27 kDa^45^. Consistent with these results, the current study presents no evidence regarding the Cry1C receptor role of CsCad. However, the knockdown of the cadherin genes *CsCad1* (GenBank accession no. AY118272, protein ID: AAM78590) and *CsCad2* (GenBank accession no. JQ747493, protein ID: AGG36450), which differ from the CsCad used in this study (GenBank accession no. DQ821523, protein ID: ABG91735), resulted in a decreased susceptibility to Cry1C in *C*. *suppressalis*^32^. Unfortunately, the binding data of CsCad1 and CsCad2 with Cry1C were not showed in this study^32^. We compared the sequences of the CsCad-CR11-MPED regions, as critical toxin-binding regions, and the full lengths of the three CsCads and found 99.67% identity (difference in a single amino acid) in the CsCad-CR11-MPED regions (Supplementary Fig. S1) and 97.78% identity in the full lengths sequences between our CsCad (ABG91735) and CsCad1 (AAM78590). However, only 14.33% and 6.68% identities were found in the CsCad-CR11-MPED regions (Supplementary Fig. S1) and full-length sequences between our CsCad (ABG91735) and CsCad2 (AGG36450), respectively. Presumably, the difference in HIS^1537^ and GLU^1537^ between our CsCad and the CsCad1 sequences may be the main reason leading to the differential functional roles in Cry1Ab and Cry1C toxicity to *C*. *suppressalis*. Single amino acid mutations in Bt toxin receptor proteins from the insect midgut that reduce toxin binding and lead to Bt resistance have been reported in other lepidopteran species^16,46,47^. Certainly, further studies investigating gene knockout or gene mutations should be conducted to validate this speculation. Additionally, the intoxication by Cry toxins may be specific to selected cadherin genes^34^. For example, two cadherins in *Alphitobius diaperinus* have been shown to participate in intoxication by Cry11Ba and Cry4Ba^42,48^.

Overall, the results in this work provide the first evidence that CsCad plays differential functional roles in Cry1Ab and Cry1C intoxication in *C*. *suppressalis*. Further work is needed to identify the Cry1C receptors in *C*. *suppressalis*. The lack of shared receptors between Cry1Ab and Cry1C supports the hypothesis that Cry1C is a candidate gene for the development of pyramided insect-resistant transgenic rice with Cry1A toxin for the control of *C*. *suppressalis* and rice pest complexes and reducing the risk of insect resistance evolution.

## Materials and Methods

### Insects and Bt toxins

A laboratory colony of *C*. *suppressalis* was initiated from larvae collected from paddy fields in Beijing (P. R. China) in 2009. The insects were reared on an artificial diet as previously described^49^ without exposure to any Bt toxins for 10 generations before use. All cultures were kept under constant conditions (27 ± 1 °C, 70–80% RH, and a photoperiod of 16:8 h light:dark).

Trypsin-activated Cry1Ab and Cry1C toxins purified from recombinant *Escherichia coli* cultures were provided as lyophilized powders by Marianne Pusztai-Carey (Case Western Reserve University, Cleveland, OH, USA).

### Expression profiles of *CsCad* during different developmental stages in different tissues

Larvae (first-, second-, third-, fourth- and fifth-instar), pupae, and female and male adults were used to analyse the spatiotemporal expression patterns of *CsCad* during different developmental stages. The head, thorax, abdomen, foregut (FG), midgut (MG), hindgut (HG), fat body and whole body without gut tissue (WBWG) of fourth-instar larvae were used for the analysis of the tissue-specific *CsCad* expression. The total RNA was extracted from each sample using TRIzol (Invitrogen, Carlsbad, CA, USA) according to the manufacturer’s instructions. cDNA was synthesized for each sample using reverse transcription PCR (RT-PCR) with a FastQuant RT Kit (with gDNase) (Tiangen Biotech, Beijing, China). The abundance of *CsCad* mRNA in each sample was detected by quantitative real-time PCR (qPCR) using Maxima Probe/ROX qPCR Master Mix (2×) (Thermo Fisher Scientific, Beijing, China) and the ABI Prism 7500 Fast Detection System (Applied Biosystems, Foster City, CA, USA). Two housekeeping genes from *C*. *suppressalis*, i.e., *β-actin* and Elongation Factor (*EF*), were selected as internal controls, and the primers of all three genes for qPCR (Table S1) were designed with Beacon Designer 7 software (Premier Biosoft International, Palo Alto, CA, USA) based on the full-length sequence of *CsCad* deposited in GenBank (accession no. DQ821523). This *CsCad* sequence was cloned from the midgut of *C*. *suppressalis* collected from a paddy field in Beijing, and the preliminary functional role of CsCad in Cry toxin intoxication was shown in Yu *et al*.^30^. The reactions for each sample (15 μl) consisted of 7.5 μl of Master Mix (2×), 1.0 μl of sample cDNA (100 ng/μl), 0.1 μl of probe (10 μM), 0.15 μl of each forward and reverse primer (10 μM), and 6.1 μl of sterilized ultrapure H_2_O (Millipore, Massachusetts, USA). The cycling parameters included incubation for 2 min at 50 °C, an initial denaturation step at 95 °C for 10 min, and 40 cycles of 95 °C for 15 s, 60 °C for 30 s and 72 °C for 30 s. The qPCR reactions were conducted with three technical replicates of each of three independent biological samples. The relative expression levels were analysed using the comparative 2^−ΔΔCT^ quantitation method, and the transcript levels of the first-instar larvae (for comparisons across life stages) and larval FG tissue (for comparisons across tissues) were standardized to 1. A one-way ANOVA with Holm-Sidak test (overall significance level = 0.05) was used to determine the statistically significant differences between the treatments.

### Cloning, expression and purification of the truncated CsCad peptide fragment

The final cadherin repeat and membrane-proximal region in lepidopteran insects are the critical binding and functional domains of Cry toxin^7,11^. Accordingly, we selected the eleventh cadherin repeat and MPED region as the potential candidate region for the binding assays. A truncated CsCad fragment (CsCad-CR11-MPED) encoding the CR11 and MPED regions (residues 1262–1565) was amplified using specific primers (Supplementary Table S1) designed according to the full-length sequence of the *CsCad* gene. The PCR products were purified with a TIANgel Midi Purification Kit (Tiangen Biotech, Beijing, China) and cloned into a pEASY-E1 expression vector (TransGen, Beijing, China) following the manufacturer’s instructions. The constructed plasmid and coding sequence were confirmed by DNA sequencing (BGI Tech, Beijing, China). The plasmids were transformed into *E*. *coli* Rosetta (DE3) Competent Cells (TaKaRa Bio Inc., Dalian, China), and positive clones were selected at 37 °C on LB plates containing 100 μg/ml ampicillin (Tiangen Biotech). The CsCad-CR11-MPED peptide fragment was overexpressed as inclusion bodies by induction with 0.8 mM isopropyl ß-D-1-thiogalactopyranoside (IPTG). After a 6-h post-induction incubation at 37 °C and 200 rpm, the cells containing cytoplasmic inclusions were harvested by centrifugation at 8,000 × *g* for 10 min (4 °C), resuspended and lysed in 10 mM Tris-HCl buffer (pH 8.0) by sonication. The inclusion bodies were isolated from the crude cell lysate by centrifugation at 12,000 × *g* for 15 min (4 °C) and solubilized in dissolution buffer (8 M urea, 20 mM Tris-HCl, 0.5 M NaCl, ad 10 mM imidazole, pH 8.0) by incubation at a 200-rpm constant shaking rate for 1 h (25 °C). The recombinant protein contained a His-tag, which was used for the affinity purification with a nickel-nitrilotriacetic acid (Ni-NTA) affinity column (Proteinlso Ni-NTA Resin, Transgen); the column was eluted with 300 mM imidazole. The eluted proteins were collected and desalted with Slide-A-Lyzer dialysis cassettes (10 K MWCO, Pierce) in 10 mM Tris-HCl (pH 8.0) at 4 °C overnight. The quantity of the inclusion body protein was determined by the Bradford method^50^, and the protein quality was analysed by 10% sodium dodecyl sulphate polyacrylamide gel electrophoresis (SDS-PAGE).

### Preparation of antibody against the CsCad-CR11-MPED peptide

The purified CsCad-CR11-MPED peptide was used as an antigen to develop antisera in injected New Zealand white rabbits. Briefly, the purified CsCad-CR11-MPED peptide was emulsified with an equal volume of Freund’s complete adjuvant for the first injection and incomplete adjuvant for three additional injections. These four injections were performed at two-week intervals, and antiserum was collected after the final injection. The anti-CsCad-CR11-MPED serum was aliquoted, and the titre was measured by an enzyme-linked immunosorbent assay (ELISA)^17^. Subsequently, the specificity of the antiserum (1:5,000 dilution) to CsCad in brush border membrane vesicles of *C*. *suppressalis* was determined by Western blotting as described by Liu *et al*.^17^. The blots were developed using goat anti-rabbit IgG(H + L) HRP-conjugated secondary antibody (Transgen) (1:10, 000) and the EasySee Western Blot Kit (Transgen, CN).

### Ligand blotting

The purified CsCad-CR11-MPED peptide fragment (2.0 μg) was resolved by 10% SDS-PAGE and then transferred onto polyvinylidene fluoride (PVDF) filters (Millipore). After blocking with blocking buffer (1 × PBS buffer with 5% dry skim milk and 0.1% Tween-20, pH 7.4), the filters were probed with 20 nM Cry1Ab or 20 nM Cry1C toxins at 4 °C overnight. After the unbound toxins were washed with washing buffer (1 × PBS buffer with 0.1% BSA and 0.1% Tween-20, pH 7.4), the bound toxin was detected using a polyclonal anti-Cry1Ab antibody (Youlong Biotech Company, Shanghai, China) (1:5,000, 1 h), followed by a horseradish peroxidase (HRP)-conjugated secondary antibody (ZSGB-BIO, Beijing, China) (1:10,000, 1 h). The blots were developed using the Enhanced Chemiluminescence (ECL) Western Blot Kit (CWBIO, Beijing, China) and imaged using an ImageQuant LAS 4000 digital imaging system (GE Healthcare).

### ELISA binding assays

To determine the binding affinities of the Cry1Ab and Cry1C toxins to the CsCad-CR11-MPED peptide, an ELISA was performed as previously described^33,48,51^. The purified CsCad-CR11-MPED peptide was biotinylated using a 50-fold molar excess of sulpho-NHS photocleavable biotin (Pierce, Rockford, IL, USA) according to the manufacturer’s instructions. The final reaction was dialysed in buffer (PBS buffer, pH 7.4) to remove the non-reacted biotin. The microtitre plates (Costar Assay Plate, 96 Well, High Binding, Corning, Inc., NY, USA) were coated with chymotrypsin-activated (1.0 µg/well) Cry1Ab or Cry1C in 50 µl of coating buffer (50 mM Na_2_CO_3_, pH 9.6) overnight at 4 °C, and then the plate was blocked for 2 h with blocking buffer (0.01 M Tris-HCL, 0.5 g/litre PEG-2000, 0.5 g/litre polyvinylpyrrolidone, 1 ml/litre Tween-20, and 5 g/litre trehalose, pH 7.4). After blocking, the wells were probed with increasing concentrations of biotin-labelled CsCad-CR11-MPED peptide with or without a 1,000-fold molar excess of unlabelled CsCad-CR11-MPED peptide. After washing, the plate was incubated with horseradish peroxidase-conjugated streptavidin (SA-HRP, Pierce) and then incubated with HRP chromogenic substrate (1-Step Ultra TMB-ELISA, EnviroLogix, Inc., USA) to quantify the bound peptide. Colour development was stopped by adding 1 M hydrochloric acid, and the absorbance was measured at 450 nM using a microplate reader (BioTek PowerWave XS2, USA). The data were analysed using SigmaPlot v.12.5 software (Systat Software, San Jose, CA) to determine the best site model fit.

### Effects of the CsCad-CR11-MPED peptide on the toxicity of Cry1Ab or Cry1C to *C*. *suppressalis*

The effects of the CsCad-CR11-MPED peptide on the toxicity of Cry1Ab or Cry1C to *C*. *suppressalis* first-instar larvae were evaluated by a diet incorporation bioassay. Activated Cry1Ab (LC_20_ dose: 0.25 μg/ml) or Cry1C (LC_20_ dose: 0.35 μg/ml) was mixed with the CsCad-CR11-MPED peptide at toxin/peptide mass ratios of 1:0, 1:1, 1:10, 1:50 and 1:100 in 1 × PBS buffer (pH 7.4); the negative controls included the CsCad-CR11-MPED peptide or Cry1Ab toxin alone in 1 × PBS buffer. One hundred microlitres of each test solution was overlaid onto a 48-well plate that had been filled with 1 ml of artificial diet. After drying, one first-instar larva starved for 4–6 h was placed in each well of the plate, and 48 larvae were tested in each replicate. Four replicates were performed per treatment. The bioassay trays were maintained in an insect culture room under constant conditions (27 ± 1 °C, 70–80% RH, and a photoperiod of 16:8 h light:dark). Larval mortality was recorded after 6 days of testing.

### Silencing the expression of *CsCad* with RNA interference and its effect on the toxicity of Cry1Ab or Cry1C to *C*. *suppressalis*

Block-iT RNAi Designer (http://rnaidesigner.invitrogen.com/rnaiexpress/) was used to design 25-nucleotide Stealth small interfering RNAs (siRNA) targeting the *CsCad* genes and Stealth siRNA negative control duplexes. The sense sequences of Stealth siRNA were 5′-GGGAGCUGUUUAGACUGUCUAUAGU-3′ located from 1076 bp to 1085 bp of CsCad. Both the siRNAs and control duplexes were chemically synthesized (Invitrogen), diluted in RNA dilution buffer (10 mM Tris-HCl, 20 mM NaCl, and 1 mM EDTA, pH 8.0) and stored at −80 °C until use.

The oral delivery of Stealth siRNA was used for the RNAi in this study. Starved *C*. *suppressalis* first-instar larvae were fed a diet^49^ containing 4.7 µg/g of the specific Stealth siRNA targeting the *CsCad* gene for 48 h. Equal amounts of RNA dilution buffer and Stealth RNAi control duplexes mixed with the diet were used as blank and negative controls, respectively. The diets containing Stealth siRNA, Stealth RNAi control duplexes or RNA dilution buffer were changed each day. At least twenty-four neonates were used in the oral delivery tests of the RNAi interference. Three replicates were performed per treatment.

The total RNA was isolated from the neonates 24 h, 48 h, 72 h and 96 h post-feeding. The transcript levels of *CsCad* were evaluated via a knockdown analysis by qPCR with specific primers (Table S1) using Maxima Probe/ROX qPCR Master Mix (2×) (Thermo Fisher Scientific, Beijing, China). qPCR was performed according to the method described above.

Larvae fed Stealth RNA, negative control duplexes or buffer were used for Western blotting to determine the expression of the CsCad protein following the different treatments. The CsCad protein was extracted from the pool of 24 first-instar larvae in respective control and RNAi treatments groups by using ProteoExtract Transmembrane Protein Extraction Kit (Novagen) following the manufacturer’s instructions and used for Western blot analysis. Total soluble proteins from larvae administered different treatments were extracted for the detection of *β-actin* as a control. The procedure used for Western blotting was performed as described above.

Parallel Cry1Ab or Cry1C toxicity bioassays were conducted using first-instar larvae subjected to each RNAi treatment. Larvae previously fed on diets containing Stealth siRNA, Stealth siRNA control duplexes (negative control treatment) or RNA dilution buffer (control treatment) for RNAi for 48 h were moved to a diet of 1.25 µg/g activated Cry1Ab toxin (LC_80_ dose) or 1.35 µg/g activated Cry1C toxin (LC_80_ dose) for the bioassays. The larval mortality was determined after 6 days of testing with the Cry1Ab or Cry1C toxin diets. Twenty-four larvae were tested in each treatment, and the treatments were replicated three times. The bioassay trays were maintained in an insect culture room under constant conditions (27 ± 1 °C, 70–80% RH, and a photoperiod of 16:8 h light:dark). A one-way ANOVA with the Holm-Sidak test (overall significance level = 0.5) was performed to determine the significance of the differences among treatments.

## Supplementary information

Supporting Information
