# Supplementary material for: Cadherin CsCad plays differential functional roles in Cry1Ab and Cry1C intoxication in Chilo suppressalis
Source: Sci Rep. 2019 Jun 11;9:8507. doi: 10.1038/s41598-019-44451-5 (PMC6559963; doi:10.1038/s41598-019-44451-5)
Supplement: Supplementary file 1 — Supporting Information [file 41598_2019_44451_MOESM1_ESM.pdf]

For submission to Scientific Reports

Address for correspondence:

Lanzhi Han

Institute of Plant Protection,

Chinese Academy of Agricultural Sciences

No. 2, West Yuanmingyuan Road,

Beijing 100193, P. R. China.

E-mail: lzhan@ippcaas.cn

Cadherin CsCad plays differential functional roles in Cry1Ab and Cry1C intoxication in *Chilo suppressalis*

Lixiao Du, Geng Chen, Lanzhi Han, Yufa Peng

State Key Laboratory for Biology of Plant Diseases and Insect Pests, Institute of Plant Protection, Chinese Academy of Agricultural Sciences, Beijing 100193, China

*Correspondence: lzhan@ippcaas.cn

Supporting Information

Table S1 Primers used for the cloning and expression of *CsCad* genes from *C. suppressalis*

Name	Sequence (5'-3')	Purpose
EF-F	GCAAGGAAGGAAAGGCTGAGGGTA	End-to-end PCR amplification of the *elongation factor (EF)* gene
EF-R	CCAGGGTGGTTGAGCACAATGACT	
tq*Actin*-F	TGAGATCGTGCGCGACAT	Primers for quantitative real-time PCR (qPCR)
tq*Actin*-R	GGCCATCTCCTGCTCGAA	
tq*EF*-F	CGCTGGCGACTCCAAAA	
tq*EF*-R	CACAATGACTTGAGCCGTGAA	
tq*CsCad*-F	ACTGCTGACTTTACTACTG	
tq*CsCad*-R	CCTTGATGTTCTCGTTGTA	
tq*Actin*-P	AGAAGCTCTGCTACGTCGCCCTCGA	TaqMan probes for qPCR
tq*EF*-P	CAACCCACCCAAAGGAGCTGCAGA	
tq*CsCad*-P	TCGTGCTATCGGTCGTGTTCTC	
Truncated *CsCad* fragment-F	GGTTTGCACGGGGTCAGCGGA	Primers for amplifying the truncated CsCad-CR11-MPED peptide fragment
Truncated *CsCad* fragment-R	GCAACCGACCCCAAGAACTA	

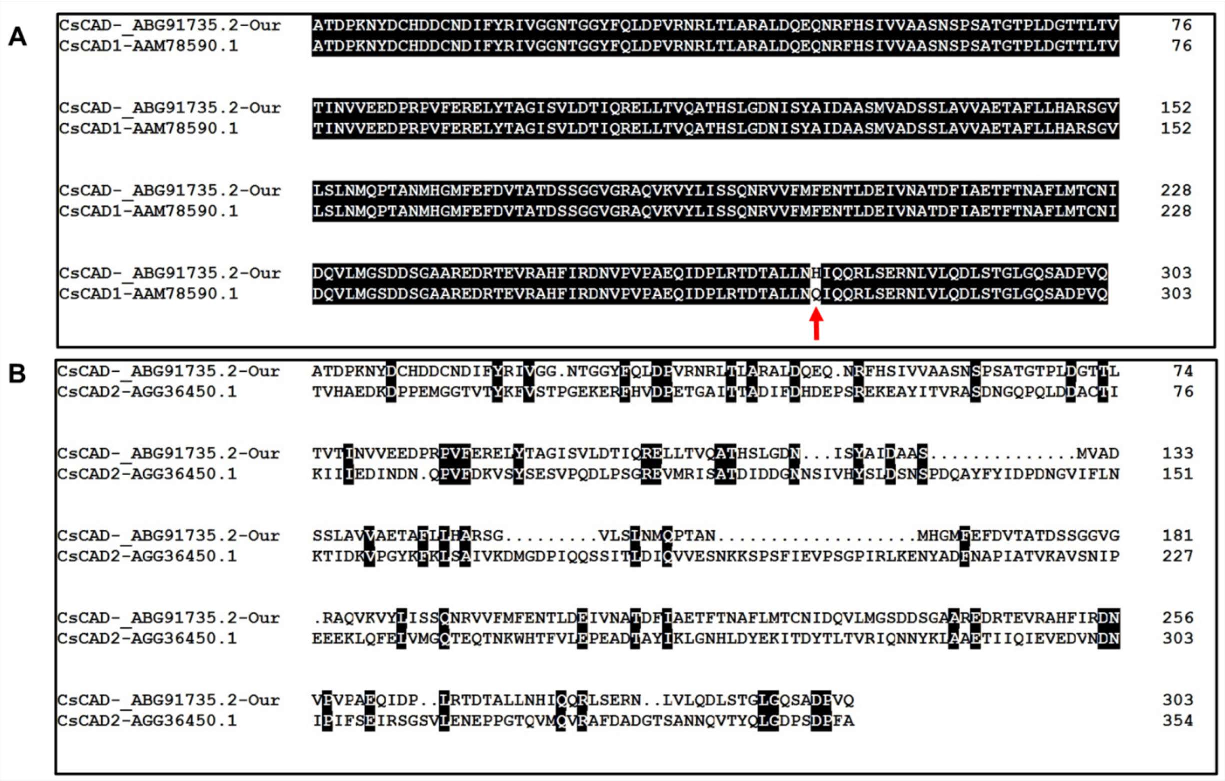

Figure S1 Alignment of the CsCad-CR11-MPED peptide fragments, including the eleventh cadherin repeat and MPED region as the critical toxin binding region, among CsCad1 (AAM78590), CsCad2 (AGG36450) and CaCad (ABG91735) used in this study. (A) Alignment of peptide fragments between CsCad1 (AAM78590) and CaCad (ABG91735); (B) Alignment of peptide fragments between CsCad2 (AGG36450) and CaCad (ABG91735).
